# Dr. William Jasper Packwood

**Published:** 1906-11

**Authors:** 


### OBITUARY.

DR. WILLIAM JASPER PACKWOOD, of Buffalo, died suddenly at his home September 24, 1906, aged 54 years. He graduated at the University of Buffalo in 1876 and had been engaged in the active practice of medicine in this city since that time.

